# Assessment of Factors Associated with Health Literacy Among Afghan Refugees in Pakistan

**DOI:** 10.3390/healthcare14081034

**Published:** 2026-04-14

**Authors:** Atta Ur Rehman, Rubeena Zakar, Gulzar H. Shah, Ume Hani, Muhammad Zakria Zakar, Tran Nguyen

**Affiliations:** 1 Department of Public Health, University of the Punjab, Lahore 54590, Pakistan; atta.rehman@ihms.edu.pk (A.U.R.); rubeenaz.iscs@pu.edu.pk (R.Z.); 2 Department of Health Policy and Community Health, Jiann-Ping Hsu College of Public Health, Georgia Southern University, Statesboro, GA 30458, USA; 3 Holy Family Hospital, Rawalpindi Medical University, Rawalpindi 46000, Pakistan; hani@ihms.edu.pk; 4 Vice Chancellor Office, University of Kotli, Azad Jammu and Kashmir, Kotli 11100, Pakistan; vc@uokajk.edu.pk; 5 School of Public Health, Department of Health Management, Economics, & Policy, Augusta University, Augusta, GA 30912, USA; trnguyen@augusta.edu

**Keywords:** refugees, health literacy, discrimination, health promotion, Pakistan

## Abstract

**Introduction:** Health literacy enables refugees to assess, understand, and utilize health information effectively. This investigation aims to identify factors influencing health literacy levels among Afghan refugees in Pakistan. **Methods:** A cross-sectional survey using a multistage sampling approach was conducted to collect data from 1185 refugees. Health literacy levels were measured using the pre-validated All Aspects of Health Literacy tool in five districts of Punjab and Khyber Pakhtunkhwa provinces that met the inclusion criteria. We used logistic regression models to analyze the dichotomous dependent variables. **Results:** A significant proportion of Afghan refugees demonstrated low functional health literacy and required assistance with reading and completing health documentation. Afghan refugees in the younger age group, male gender, higher monthly income, and access to healthcare information and clean water were more functionally literate in health. More than two thirds of the Afghan refugee population had adequate communicative health literacy with health care professionals in Pakistan. Most refugees believed that healthy lifestyles information and encouragement were more crucial for health than housing, employment, education, and local infrastructure. **Conclusions:** Afghan refugees in Pakistan lacked functional health literacy, critical health literacy, and overall health literacy. However, they have adequate communicative health literacy. This initial survey added new data on Afghans’ health literacy levels, which could help stakeholders strengthen health promotion initiatives within the healthcare system to improve health outcomes.

## 1. Introduction

Health literacy is the outcome of socio-structural context as well as individual characteristics, enabling environment, requirements, and the complexity of the healthcare system [1]. Global assessments highlighted the functional, communicative, and critical domains of health literacy. Functional health literacy assesses knowledge of the health care system through essential reading and writing skills. Communicative health literacy measures a person’s communication skills and ability to interpret information. Critical health literacy involves the socio-cognitive skills required to appraise information. Community participation may be evaluated by the society’s engagement in terms of life quality and motivation to live healthier lives [2]. The World Health Organization Commission on Social Determinants of Health has acknowledged the importance of health literacy in assessing health inequalities in low and middle-income countries [3]. Low health literacy is associated with higher rates of illness and longer hospital stays. Governments are now concentrating on enhancing their healthcare systems through health literacy promotion, as it has been recognized to improve an individual’s health status [4].

Pakistan’s healthcare system ranked 130th out of 195 countries in terms of accessibility and quality in 2021, according to the Global Health Security Index [5]. Investigations into health literacy in Pakistan reported low levels of health literacy among patients [6], adult population [7], and poor functional health literacy levels among adolescents [8]. Rural populations had lower health literacy levels compared to the country’s urban population [9]. Numerous factors were associated with the low levels of health literacy [6,7,8,9,10]. Access to the healthcare system is one of the primary needs of refugees after resettlement in their host country. Afghan refugees have added to the burdened healthcare system in Pakistan. Pakistan hosted over 1.42 million registered Afghan refugees over the last 43 years, and the refugees’ influx has reportedly increased in Pakistan since the Taliban took control of Afghanistan in August 2021 [11].

Refugee health literacy depends primarily on the host nation’s living environment and medical facilities. Linguistic and cultural challenges in the new country, with restricted access to health care services and basic necessities of life, may contribute to low health literacy levels among refugees [12]. Although younger refugees have better prospects of assimilation and success [12], low levels of health literacy resulted in deficient self-health management and poor health status, which may enhance stress and health inequity [13]. Refugees’ poor health may result from complex interactions among pre- and post-migration factors, the healthcare system, and other factors that influence health [13,14]. Refugees from Afghanistan tend to have low health literacy and a high prevalence of mental disorders globally [14]. Afghan women had the lowest level of health literacy among Asian women [15]. The functional health literacy of refugees in Europe was inadequate due to language and cultural barriers [13]. It was necessary to improve health literacy to facilitate health communication among refugees in Sweden [16]. The United States refugee populations faced significant challenges in improving health outcomes due to low health literacy and inadequate language skills [17]. Health literacy among refugees can be influenced by institutional factors, including legal assistance, language settings, administrative preparedness, and extended relocation, as well as individual abilities. In persistent refugee environments such as Pakistan, similar institutional constraints may affect the functional, communicative, and critical dimensions of health literacy, leading to a range of literacy rates despite longer stays in the host state. The conceptual notion that health literacy among refugees is influenced by both personal experiences and the broader context of relocation underpins this study. The ability of refugees to acquire, comprehend, and apply health knowledge is influenced by their socioeconomic status, including household financial resources and educational attainment, as well as their access to medical services and data. Settlement-associated variables, such as housing location and length of stay, further shape these linkages, leading to heterogeneity across many health literacy subdomains among refugee populations.

Literature-based predictors of health literacy among refugees were socioeconomic demographics, language and cultural differences, social determinants of health, healthcare professionals’ behavior, and patients’ communication skills [18,19,20,21]. Good health status is a prerequisite for a high quality of life, and health literacy is necessary to stay healthy. Refugees may have more diverse predictors and levels of health literacy than residents of their host countries [22]. Global public health development depends on the promotion of health literacy. Health literacy enables people to effectively assess, understand, appraise, and apply health-associated data to achieve improved health outcomes [23]. Health literacy gaps between the host and refugee populations may lead to social injustice and health inequity [22]. Every refugee in the host society needs to be health-literate enough to make appropriate healthcare decisions for themselves and their family members. Better health literacy among refugees in the community requires support from host countries. Highly health-literate individuals were more likely to use public health preventive measures to improve health outcomes [24]. Studies based on large-scale primary data on the health literacy of Afghan refugees are scarce despite more than 45 years of refugee status in the host country. Therefore, we initiated this quantitative study comprising a large sample of participants. The study aims to assess predictors of health literacy across functional, communicative, critical, and empowerment-related domains among Afghan refugees in Pakistan.

## 2. Materials and Methods

### 2.1. Study Design and Sampling Approach

A cross-sectional study design with multistage sampling was used to collect data from refugees. The provinces of Khyber Pakhtunkhwa and Punjab were included in this investigation because the majority of the refugee population resided there, to allow female participation and to ensure representation of educated people. District-wise clusters were chosen from the provinces in the first stage of sampling based on the participation of both genders in the pilot study. Haripur, Mardan, Peshawar, and Nowshera were the districts selected from Khyber Pakhtunkhwa, while Kot Chandana (District Mianwali) was included from Punjab in the sampling frame. Afghan male refugees did not allow females to participate in this investigation in other provinces and districts due to sociocultural constraints. The second sampling stage employed proportionate sampling to estimate the required sample size based on the refugee population in each selected district. Organizations working with Afghan refugees do not share information about refugees (list). Thus, systematic random sampling was used in the third stage to complete the required sample size. The second refugee household from the first ten (k) was selected using a random number generator, and the appropriate sample size was determined by adding (k) at regular intervals.

Refugees with proof of Afghan status, as reflected on their registration card, and the presence of both genders in the household were included from provinces and districts with Afghan representation. The investigation included refugees residing in the selected district during the data collection period and who were willing to participate. Refugees who were unable to understand consent procedures or respond to a survey questionnaire were excluded from the study. However, this approach may have contributed to gender as well as geographical bias and limited the outcome’s generalizability, especially for women in more constrained circumstances and undocumented refugees.

### 2.2. Calculation of Sample Size

The sample size of 1185 was calculated using the known population for the sampling formula *n* = N/(1 + N [e]^2^). The total number of refugee families (N = 35,082) and sampling error (e ± 0.03) were used in the formula [25,26]. The probability of rejection, estimated at 10%, was added to the final sample size. The respondents were selected from each province and district using a proportionate random sampling formula.

### 2.3. Data Collection Tool

The data collection instrument comprised a pre-validated questionnaire. Several instruments are available to assess health literacy, including the newest vital sign, Test of Functional Health Literacy in Adults, Rapid Estimate of Adult Literacy in Medicine, Wide Range Achievement Test, Health Literacy Skills Instrument, Health Literacy Management Scale, Public Health Literacy Knowledge Scale, and the European Health Literacy Survey Questionnaire. However, the All Aspects of Health Literacy Scale was selected because it uniquely captures functional, communicative, critical, and empowerment-related domains in a reliable, valid, and field-feasible manner. Based on the needs of the local community, factors influencing health literacy were identified from the Syrian Refugee Survey [27]. The All Aspects of Health Literacy Scale (AAHLS) is a validated instrument with acceptable psychometric properties, including internal consistency [2]. For this study, the scale was translated and cross-culturally adapted into Pashto and Dari using bilingual forward–backward translation, contextual review, and pilot testing among Afghan refugees to ensure cultural relevance and comprehension. The All Aspects of Health Literacy Scale was used, with a Cronbach’s alpha of 0.74. The authors provided consent to the use of the tools in Pakistan. The tool scale consisted of 14 items measuring four aspects: using written health information, communicating with health care providers, health information management, and appraisal and assertion of individual autonomy regarding health [2]. Following the recommendations of the World Health Organization, bilingual Afghan refugee research associates were hired to translate Pashto and Dari and to collect data. The Syrian Refugee Survey was used to construct socio-economic and demographic variables and factors predicting health-associated quality of life-based on the requirements of the local population [27]. Of the 2137 individuals invited to participate in the study, 1185 completed the survey, yielding a response rate of 55.45%.

### 2.4. Study Variables

#### Dependent Variable

This study comprises 14 dependent variables grouped into four categories: (1) functional health literacy, (2) communicative health literacy, (3) critical health literacy, and (4) empowerment related to health literacy. The first category, functional health literacy, includes three variables measured by the following questions: “Do you need help interpreting medical information provided by a doctor, nurse, or pharmacist?” “Can you easily access someone to assist you if you need help?” “Do you need assistance completing official documents?” The communicative health literacy category comprises three variables assessed through the question: “Do you provide all the necessary information when you talk to a doctor or nurse?” Do you ask the questions you need to ask when you talk to a doctor or nurse? Do you ensure that they explain anything that you do not understand when you talk to a doctor or nurse? The third group of dependent variables comprises four variables derived from the question: “Are you someone who likes to find out a lot of different information about your health?” How often do you think carefully about whether health information makes sense in your particular situation? How often do you try to determine whether information about your health can be trusted? Are you the sort of person who might question your doctor or nurse’s advice based on your research? The final group includes four variables assessed by the question: Do you think there are many ways to have a say in what the government does about health? Have you taken action to do something about a health issue affecting your family or community in the last 12 months? Do you think information and encouragement to lead healthy lifestyles matter most for everyone’s health? Do you think good housing, education, decent jobs, and good local facilities matter most for everyone’s health? The answer choices for these items were often, sometimes, rarely, or never, which were recorded as dichotomous variables: yes (often and sometimes) and no (rarely and not applicable).

### 2.5. Independent Variables

The study’s independent variables included sociodemographic characteristics and hypothesized predictors of health literacy.

The sociodemographic characteristics include age (in years), gender (male or female), marital status (married, unmarried, or widowed/separated), family setup (joint family system, or nuclear), province of resettlement (Khyber Pakhtunkhwa, Haripur, Mardan, Nowshera, Peshawar, Punjab, or Mianwali), mother language (Dari, Pashtu, or other), number of years in the host country (<10 years, 10–19 years, or >20 years), residence type (owner, rent, or donor/government supported), place of residence (urban or rural), level of education (uneducated, <10 years of education, 10–14 years of education, or 16 years of education and greater), employment status (employed or unemployed), family monthly income (<25,000, 25,000–50,000, 50,001–75,000, or >75,001), and self-rated socioeconomic status (high, average, or low).

The hypothesized predictors of health literacy comprise substance abuse from smoking (frequently, occasionally, or non-smoker), substance abuse of Naswar (frequently, occasionally, or non-user), cultural compatibility (always, sometimes, or never), linguistic barriers (always, sometimes, or never), face discrimination (always, sometimes, or never), social inclusion (always, sometimes, or never), socioeconomic support (always, sometimes, or never), access to clean water (always, sometimes, or never), access to sanitation (always, sometimes, or never), access to education (always, sometimes, or never), access to healthcare information (always, sometimes, or never), access to healthcare (always, sometimes, or never), current health status (healthy or sick), chronic health illness (yes or no) and last time visited health facility (within last 1 month, within last 1 year, more than 1 year).

### 2.6. Data Analysis

IBM SPSS Statistics version 24.0 (IBM Corporation, Armonk, NY, USA) was used for all data analyses. Descriptive statistics were computed for all independent and dependent variables as appropriate. Logistic regression analysis was used to examine the association between the dependent and independent variables. Both simple logistic regression and multivariate logistic regression analyses were conducted to identify significant predictors while controlling for potential confounding factors.

Prior to performing the regression analysis, the assumptions of logistic regression were assessed. Multicollinearity among the independent variables was examined using the variance inflation factor (VIF), and no significant multicollinearity was detected. The independence of observations was ensured by the study design, and the outcome variable was binary, satisfying a key requirement for logistic regression.

Fourteen multivariable logistic regression models were performed for each dependent variable, and all independent variables were included in each model. This approach allowed the effect of each independent variable to be estimated while controlling for the influence of the other variables in the model, thereby providing adjusted estimates and reducing the potential for confounding. Model performance and fit were evaluated using diagnostic statistics available in the SPSS logistic regression procedure. These included the Hosmer–Lemeshow goodness-of-fit test (with *p*-values greater than 0.05), the omnibus test of model coefficients (with *p*-values less than 0.05), and pseudo-R^2^ measures (Cox–Snell, ranging from 0.037 to 0.067, and Nagelkerke, ranging from 0.055 to 0.108). Overall, the results indicated that the logistic regression models demonstrated acceptable fit, with pseudo-R^2^ values within ranges commonly observed in applied logistic regression analyses.

### 2.7. Ethical Approval

The University of the Punjab Advanced Studies and Research Board granted ethical approval for this survey on 16 January 2020, via notification number D.NO1950ACAD. The Afghan refugee in charge of the area, known locally as Malik, also gave his written consent for the project. Afghan refugees were made aware of the importance and objectives of the research. Written informed consent was obtained from refugees before filling out the survey questionnaire. All refugees received information about their voluntary contribution and assurances regarding the anonymity and privacy of their data. The questionnaires were coded anonymously, and no personal information, such as names, telephone numbers, or addresses, was collected to ensure participants’ privacy.

## 3. Results

### 3.1. Descriptive Statistics About the Socio-Demographic and Other Characteristics of the Afghan Refugees

The refugee majority was in the 46–60-year age group (39.2%). Most Afghan refugees were males (51.7%), married (55.4%), and living in the joint family system (74.8%). The Khyber Pakhtunkhwa province accounted for 93.2% of the respondents among the relocated Afghan refugees, whereas the Punjab province accounted for 6.8%. Pashtun was the mother tongue of almost half of the refugees (50.5%). The majority of refugees (57.9%) have been living in the host state since 2002, compared to 33.1% who arrived before 2012 and only 9% who have been resettled in the past ten years. Urban areas were the primary residence for most refugees (59.9%), and the majority of households were supported by donors (77.8%). The survey respondents comprised almost half of the refugees (54.6%) with less than 10 years of education. Employed respondents were 47.2% in this investigation. The family’s monthly income in Pakistani rupees was less than 25,000 for most of the refugees (69.7%). Only 2.7% of the informants’ self-reported socioeconomic position was high, compared with nearly two-thirds who reported low status.

A slim majority (50.8%) of the sampled population were non-smokers, and 57.6% had never misused a substance (Naswar) in their lifetime. Almost two-thirds of the refugees (74.9%) were culturally compatible with the host country. More than half never experienced linguistic barriers (54.2%) and discrimination (53.5%) in the host country. The majority of refugees (56.9%) reported sometimes feeling socially included despite being refugees in the host nation. Nearly two-thirds (74.6%) of the participants always received socioeconomic aid from donor agencies or the government. According to the Afghan refugees, 56.4% always had access to clean water, 57.1% always had access to sanitation, 57.2% always had access to education, and 49.5% always had access to healthcare. Of the participants, 48.9% were in good health, 38.1% had a chronic condition, and 28.2% had visited a hospital in the previous month. The socio-demographic information and health literacy predictors of Afghan refugees in Pakistan are shown in Table 1.

### 3.2. Health Literacy Among Afghan Refugees in Pakistan

Descriptive results showed limited functional health literacy, with 76.0% of participants needing help to understand information from healthcare professionals and 80.0% needing help with official documentation, although 82.2% reported that assistance was easy to find. In contrast, communicative health literacy appeared comparatively strong, as most participants reported providing information to healthcare professionals, asking needed questions, and requesting clarification when they did not understand. Critical health literacy was lower, with fewer than half of respondents reporting active searching, appraisal, or verification of health information. For empowerment-related health literacy, most participants believed there were ways to influence government action on health care, but fewer reported taking action on health issues affecting their family or community. A majority also considered information and encouragement for healthy lifestyles more important for health than housing, education, jobs, and local facilities. Table 2 presents the full item-level results. Table 2 presents the results of the scale measuring All Aspects of Health Literacy among Afghan refugees in Pakistan, including Functional health literacy (FHL), communicative health literacy (COMHL), critical health literacy (CRHL), and empowerment-related health literacy (EMP).

### 3.3. Factors Associated with Health Literacy Among Afghan Refugees in Pakistan

Logistic regression results present the factors associated with health literacy among Afghan refugees in Pakistan, as demonstrated in Table 3, Table 4, Table 5 and Table 6, and presented as adjusted odds ratios (AORs). 

#### 3.3.1. Functional Health Literacy

##### Need Help to Understand Information

Table 3 presents the factors associated with dependent variables measuring functional health literacy. Among the independent variables examined, family monthly income (measured in Pakistani rupees) was significantly associated with the need for help in understanding health-related information. Specifically, individuals from families with the highest income level (greater than 75,001 rupees per month) had significantly lower odds of needing assistance compared to those from families earning less than 25,000 rupees monthly (AOR = 0.385).

##### Easy to Find Someone for Assistance

Age, access to clean water, and access to healthcare information were significantly associated with the ease of finding assistance. Individuals aged 61 years and above had lower odds of finding assistance compared to the 18–30-year age group. Similarly, individuals who only sometimes had access to healthcare information were significantly less likely to find assistance compared to those who never had access (AOR = 0.067). In contrast, individuals who sometimes had access to clean water had significantly higher odds of finding assistance compared to those who always had access (AOR = 12.087).

##### Need Help with Official Documentation

Gender is the only variable that showed a significant association between needing help with official documentation and health literacy. Females had higher odds (AOR = 1.675) than males of needing help with official documentation.

#### 3.3.2. Communicative Health Literacy

##### Provide Information for Assistance

Among the independent variables tested, self-rated socioeconomic status (SES) and substance abuse (Naswar) showed significant associations with providing information for assistance (Table 4). Compared with the upper SES level, those in middle and lower SES levels had higher odds (AOR = 3.846 and AOR = 3.881) of providing information for assistance. The lower the self-rated SES, the higher the odds. Substance abuse (Naswar) also showed a significant association with providing information for assistance. Occasional substance abusers had higher odds (AOR = 2.804) association with providing information for assistance compared with the frequent substance abusers, while the non-users showed no significant association.

##### Inquire About the Queries

Resident type and access to education are significantly associated with inquiring about the queries. Donor/government support and rent had lower odds (AOR = 0.114 and AOR = 0.130, respectively) of inquiring about the queries compared with homeowners. Those who sometimes had access to education had lower odds (AOR = 0.592) of inquiring about the queries than those who always had access to education. In contrast, those who never accessed education showed no association.

##### Insist on Explaining Not Clear Content

Providence of resettlement and substance abuse (Naswar) showed significant associations with insisting on explaining unclear content. Compared with Haripur, Mardan had higher odds (AOR = 3.270) of insisting on explaining unclear content, while those settled in other providence showed no association. Substance abuse (Naswar) also showed a significant association with insisting on explaining unclear content. Occasional substance abusers and nonusers had higher odds (AOR = 2.927 and AOR = 1.812) of insisting on explaining unclear content compared with those who did not.

#### 3.3.3. Critical Health Literacy (CRHL)

##### Like to Search for Facts Related to Health

Table 5 presents the predictors of variables measuring critical health literacy. Gender, marital status, current health status, and chronic health conditions demonstrated significant associations with the search for health-related facts. Females have lower odds (AOR = 0.417) of searching for health-related information than their counterparts. In marital status, the widowed/separated had higher odds (AOR = 2.222) of searching for health-related facts compared with the unmarried, while the married group showed no significant association. Participants with acute illness had higher odds (AOR = 2.262) of searching for health-related facts than those who were healthy. Conversely, those with chronic conditions had lower odds (AOR = 0.430) of searching for health-related facts, compared to participants without any chronic condition.

##### Information Appropriate for the Situation

Employment status and smoking are significantly associated with the provision of appropriate information for the situation. Unemployment had higher odds of (AOR = 1.575) than employment. Non-smokers had higher odds (AOR = 2.300) of providing information appropriate to the situation than frequent smokers, while occasional smokers showed no association.

##### Evaluate the Healthcare Information Reliability

Gender, family setup, resettlement location, and social inclusion as a refugee in the host country were significantly associated with the reliability of healthcare information. Females had higher 1.978 and AOR 1.506) than their counterparts in evaluating the reliability of healthcare information. In contrast, nuclear families had lower odds (AOR = 0.653) than joint family systems for assessing the reliability of healthcare information. Mardan, Nowshera, Peshawar, and Kot Chandana Mianwali had higher odds (AOR = 3.270, AOR = 1.790, AOR = 2.486, and AOR = 4.540, respectively) than Haripur in evaluating the reliability of healthcare information. Those who sometimes and never had social inclusion as a refugee in the host country showed higher odds (AOR = 1.978 and AOR = 1.980, respectively) in evaluating the reliability of healthcare information than those who always had.

##### Inspect the Healthcare Personnel’s Advice Based on Your Own Research

Age group and social inclusion as a refugee in the host country showed significant associations with inspecting the healthcare personnel’s advice based on your own research. The oldest age group of 61 years and above had lower odds (AOR = 0.420) of inspecting the healthcare personnel’s advice based on your own research compared with the youngest age group of 18–30 years. Those who never had social inclusion as a refugee in the host country showed lower odds (AOR = 0.522) of following the healthcare personnel’s advice based on their own research than those who always had.

#### 3.3.4. Empowerment Related to Health Literacy (EMP)

##### Trust That There Are Ways to Guide Government Actions for Healthcare

Table 6 depicts the predictors of variables measuring empowerment related to health literacy. Education level and linguistic barriers in the host country were significantly associated with trust in the existence of ways to guide government actions in healthcare. Those with <10 years of education had higher odds (AOR = 1.879) than the uneducated of trusting that there are ways to guide government actions in healthcare. Other education levels showed no association. Also, those who never had linguistic barriers in the host country showed higher odds (AOR = 1.755) in trusting that there are ways to guide government actions for healthcare than those who always had. While those who sometimes had linguistic barriers in the host country showed no association.

##### Measure Taken Last Year to Tackle Health Concerns in the Community

Family setup, educational level, family monthly income (in Pakistani rupees), and chronic health illness demonstrated significant associations with measures taken last year to tackle a health concern in the community. Nuclear families had higher odds (AOR = 1.421) than joined family systems in measures taken in the previous year to tackle a health concern in the community. Those with <10 years of education had higher odds (AOR = 1.354) than the uneducated of being involved in measures taken last year to address a community health concern. Other education levels showed no association. Compared with families with monthly incomes of less than 25,000 rupees, families with the highest income level (greater than 75,001 rupees a month) had higher odds (AOR = 2.518) of having taken measures last year to tackle a health concern in the community. In contrast, those with chronic illnesses had lower odds (AOR = 0.626) than their counterparts of measures taken last year to tackle a health concern in the community.

##### Information and Encouragement to Lead Healthy Lifestyles Are Essential for Everyone’s Health

Only access to sanitation shows a significant association with information and encouragement to lead healthy lifestyles, which are essential for everyone’s health. Those who never had access to sanitation had lower odds (AOR = 0.549) of receiving information and encouragement to lead healthy lifestyles, which are essential for everyone’s health, than those who always had access to sanitation.

##### Good Housing, Education, Decent Jobs, and Good Local Facilities Are Essential for Everyone’s Health

The provinces of resettlement and cultural compatibility in the host country are significantly associated with good housing, education, decent jobs, and good local facilities essential for everyone’s health. Kot Chandana Mianwali had higher odds (AOR = 1.991) than Haripur for good housing, education, decent jobs, and good local facilities, all of which are essential for everyone’s health. Those who sometimes had cultural compatibility in the host country had higher odds (AOR = 1.469) of good housing, education, decent jobs, and essential local facilities for everyone’s health than those who always had cultural compatibility in the host country. In contrast, those who never had cultural compatibility in the host country showed no association.

## 4. Discussion

This survey uses a cross-sectional design with the pre-validated AAHL tool to examine factors influencing functional, communicative, critical, and empowerment health literacy among Afghan refugees in selected districts of Punjab and Khyber Pakhtunkhwa, addressing a historical gap in health literacy assessment. The World Health Organization reported that refugees had lower health literacy levels than those in the host countries due to their limited access to educational and health communication opportunities [28]. There is a scant amount of research literature on health literacy in Pakistan’s general population, and there is not even a single assessment of health literacy related to Afghan refugees. We therefore conducted a general health literacy comparison, as it was inappropriate to do so in detail for regions that do not host refugees or for assessment tools that differ [6,8,29]. Systematic reviews of health literacy have documented the difficulty of comparing results across studies due to differences in measurement tools and comparison methods [30,31,32]. The lack of national and international studies on the health literacy of Afghan refugees made it difficult to compare levels. The reference was established with refugees from Syria, Afghanistan, and other countries who migrated to Europe, Canada, and the United States of America due to the availability of literature [13,16,17,22,33,34]. All aspects of the health literacy tool did not specify a cutoff for high versus low health literacy. Therefore, we also considered dichotomizing the health literacy score to be suboptimal due to the complexity of the phenomenon.

Refugees’ awareness of and performance in all areas of functional, communicative, and critical health literacy are key indicators of their level of health literacy. Functional health literacy levels account for a person’s basic capacity to read and complete medical documents. Refugees in Pakistan reflected limited functional health literacy levels, just like the host adolescents in Islamabad [8] and the Afghan refugees in Sweden [13]. A significant percentage of refugees in Punjab and Khyber Pakhtunkhwa required assistance with reading and completing health documentation. Health literacy and education are interrelated, as evidenced by previous studies conducted in Europe, which found that participants with higher education levels performed better across all three domains of health literacy [35,36] and were either illiterate or educated below the 10th-grade level in this survey. Inadequate functional health literacy was also reported among non-Afghan refugees in Canada and the USA [33,34]. Higher family monthly income was linked to a reduced need for assistance in understanding health-related information among Afghan refugees in this investigation, as well as in global literature [37,38]. Refugees aged 60+ were less likely than younger age groups to find someone to assist them. A weak community support system was also identified in an international systematic review of refugees [39]. Afghan refugees’ ability to access health care information is greatly affected, as those without access are more inclined to seek assistance. Individuals who have trouble accessing health information often depend on relatives, friends, fellow citizens, and health care professionals to support them in understanding and making decisions [40]. Participants in the investigation who had access to clean water were less likely to require assistance than refugees who had access to clean water only rarely. Polluted water usage in communities was linked with low literacy [41]. Males were more functionally literate in health than females in this study. Females had higher odds than males of needing help with official documentation. Afghan women had the lowest levels of health literacy in Asia [15] because, in Afghanistan, decisions about health and education are made by the male head of household, leading to similar trends in the host country [42]. In contrast to our findings, a meta-analysis of 22 studies on refugees concluded that females had better health literacy than males [43].

Communicative health literacy was defined as patients’ communication skills during health care consultations. Refugees may be encouraged to improve their health status via effective communication within the healthcare system [44]. More than two thirds of the Afghan refugees have adequate communicative health literacy with health care professionals in Pakistan, which was almost similar to the population who never or sometimes faced linguistic barriers in the host country. Afghan refugees with low communicative health literacy scores may be attributed to the linguistic barriers and lack of language interpreters in Pakistan health care facilities for refugees in the sampled population. Limited language proficiency in the United States of America was associated with low health literacy [34]. Global research also confirmed that refugees’ limited language proficiency and lack of translators posed challenges to effective health communication [12]. The majority of Afghan refugees with higher self-rated socioeconomic status need to provide less or no information for assistance compared with middle-class and lower-class persons. The literature also recognized that individuals with higher socioeconomic status require little or no help in health care interactions [37,45]. Afghan refugees living in their own houses were more engaged in health communication and asked more questions than those living in rented or donor- or government-supported camps. Social isolation in the camps/resettlement areas may significantly influence interactions, as reported among refugees in Germany, Bangladesh, Kenya, and high-income countries, leading to lower health service utilization [46,47,48]. More educated Afghans ask more questions during health care interactions in Pakistan than less educated Afghans. The increased ability to interact with healthcare professionals and to express health concerns was substantially correlated with education [49]. Area of relocation significantly influences Afghan refugees’ communication skills, as resettlement in Mardan was associated with greater insistence on clarifying ambiguous content, as evidenced by the higher quality of life of Afghan refugees in an earlier investigation [50]. Drug addiction among refugees impairs their ability to coordinate and communicate effectively [51], also reinforced by this survey, as non-users and occasional substance abusers insisted on clarification in contrast to frequent abusers.

Communities and the general public are empowered by critical health literacy to evaluate health-related information critically and to take a more active part in social and political decisions that affect their health [52]. Critical health literacy was generally low among the sample population of refugees. Refugees’ health seems to be deteriorating due to their limited access to the healthcare system in many host countries. International research has established a direct correlation between poor levels of health literacy and refugees’ restricted access to healthcare facilities [53]. The socioeconomic status of the population affects the availability of essential tools like computers, mobile devices, and the internet, which are necessary to evaluate healthcare information [54]. Almost 2/3 of the Afghan refugees in Pakistan reported low socioeconomic status; therefore, it was difficult for them to have access to critical health literacy via information and communication technology. A critical body of research for health-related facts was more reported among male Afghans, although the reliability of healthcare information was the opposite of the sampled proportion. Low critical health literacy among female refugees may be related to the fact that over 90% of them lacked a high school certificate or were illiterate in this survey. The majority of patient education resources available were written at higher levels of English, making them incomprehensible to most uneducated and less educated populations [55]. Inadequate CHL [comprehensive health literacy] was more in separated refugees in global research in comparison to married and unmarried refugees in contrast to the finding of this investigation [56]. Health status of refugees incited the fact that ill refugees were inclined more about health incisive behaviors, but chronic patients were less concerned, as evident from earlier literature [57]. Unemployed Afghans and non-smokers considered information more appropriate; nevertheless, employment correlates with reliable health data [58]. The value of joint family system in evaluating and communicating reliable healthcare information was also evident from this research [59]. Afghans living in rural zones (Haripur camps) and socially involved demonstrated less ability to evaluate healthcare information in comparison to urban areas and less socially involved also aligned with international literature [60,61]; although, the socially included cohort better inspect the healthcare personnel’s advice based on their own research. Younger Afghan refugees better linked the information as digital competencies were more noticed in early adulthood refugees at international level [62].

Empowerment remains vital for improved health outcomes and increased health literacy. A sufficient level of critical health literacy is a prerequisite for empowerment, which can increase the involvement of refugees in making healthcare decisions [63]. The majority of Afghan refugees (58.3%) in Pakistan believed that there were multiple possibilities for influencing government health care decisions. The concept of the Afghan Loya Jirga to settle the issues may be related to the majority opinion being in favor of the government despite the low levels of critical health literacy [64]. Afghans with primary and secondary education and who never faced linguistic barriers in Pakistan shown increased trust in government, as linguistic integration remain linked with improve health outcome among refugees [65]. Refugees’ involvement in community health initiatives improves their level of awareness and increases their access to health-care services. More than half of refugees have not participated in any efforts to solve health issues over the past many years. This outcome aligns with earlier literature indicating limited community participation of refugees in host communities [66]. Nuclear Afghan families, with minimal formal education, without chronic disease took better measures last year to tackle a health concern in the community. An earlier investigation in Australia reported that healthy and educated refugees were more engaged in health promotion initiatives [67].

The majority of refugees believed that healthy lifestyles information and encouragement were more crucial for health than housing, employment, education, and local infrastructure. This result is consistent with earlier research demonstrating that many individuals consider personal behaviors (better sanitation facilitates in this research) to have a stronger relationship to healthcare outcomes than infrastructure variables. However, the presence of good infrastructure has a significant effect on an individual’s ability to engage in healthy activities [68,69]. Geographic variation in responses was observed among Afghan refugees, as living in a more developed province Punjab (Kot Chandana, Mianwali) and being intermittently culturally aligned showed likelihood for good housing, education, decent jobs, and good local facilities. Empirical evidence in the literature also supported the notion that refugees living in developed urban slums had better welfare behavioral approaches [70].

The findings of the snapshot survey revealed that low levels of health literacy among Afghan refugees were aligned with low levels of health literacy in the host nation and significantly correlated with a few health literacy predictors.

## 5. Strengths and Limitations

The findings of this study should be understood within the context of its limitations. Some of the limitations are those inherent in cross-sectional analysis. For instance, our cross-sectional study does not lend itself to making causal connections between the independent and the dependent variables. The study may also be affected by the selection bias. A potential limitation of this study is that refugees who were unable to understand the study procedures were excluded from participation, which may have introduced selection bias by underrepresenting individuals with lower levels of health literacy and could therefore affect the generalizability of the findings to the broader refugee population. Health literacy varies by context, so it is important to exercise caution when generalizing the results of this investigation. Another limitation is that the study relied on self-reported information collected during the COVID-19 pandemic, which may not fully reflect actual behaviors. In addition, some regression estimates were accompanied by relatively wide confidence intervals, which likely reflect smaller subgroup sizes for some categories. M covariates were included in the adjusted models; therefore, these estimates should be interpreted with appropriate caution. Despite these limitations, the study has several strengths that underscore the worth of our study. The study uses the data that is the first of its kind covering Afghan refugees to measure health literacy and its associated factors using the validated All Aspects of the Health Literacy tool. The refugee population in our sample characterized substantial heterogeneity across several socio-demographic characteristics. Regardless of these limitations, our study had several strengths, which include the use of validated measurement tools and a relatively large sample. Our sample of study participants also included women from a socioculturally conservative population group. We also conducted the interviews in the participants’ primary languages, Dari and Pashtu. We engaged trained native speakers to assist with data collection in order to include refugees with limited formal education.

## 6. Conclusions and Recommendations

This study provides context-specific research evidence that health literacy among Afghan refugees in Pakistan remains limited in several important domains. This is particularly true for functional, critical, and empowerment-related health literacy, although communicative health literacy appeared relatively stronger. Our study findings imply that improving refugee health outcomes will require more than access to services alone. Effective interventions will also require clearer health communication, culturally and linguistically appropriate information, and educational strategies that strengthen refugees’ ability to understand, evaluate, and use health information. The findings may help inform public health agencies, humanitarian actors, and healthcare providers working with refugee populations in designing more responsive health promotion efforts. Given the cross-sectional design and contextual limitations of the study, further research with broader, more representative samples is needed to better understand the determinants of health literacy and to inform targeted interventions. There is a need for further qualitative and quantitative research with a large, representative population to identify significant predictors of health literacy and improve access to health information and healthcare utilization.

## 7. Contribution to the Field

Refugees have a basic right to good health care, and health literacy remains vital for the better health of refugees. Investigations on the health literacy of refugees were scarce in Pakistan, despite hosting refugees since the country’s inception. This survey on Afghan refugees in Pakistan is pertinent to the need for global health care for refugees, as the health literacy evaluation presents the existing situation to recommend actions to support the protection of refugees ‘and the general public’s health in society.

## Figures and Tables

**Table 1:** Sociodemographic characteristics and predictors of health literacy among Afghan refugees in Pakistan (N = 1185).

Sociodemographic Characteristics	*n* (%)	Predictors of Health Literacy	*n* (%)
Age (in years) 18–30 31–45 46–60 >61	350 (29.5) 281 (23.8) 465 (39.2) 89 (7.5)	Substance abuse (Smoking) Frequently Occasionally Non-smokers	427 (36.0) 156 (13.2) 602 (50.8)
Gender Male Female	613 (51.7) 572 (48.3)	Substance abuse (Naswar) Frequently Occasionally Non-users	348 (29.4) 154 (13.0) 683 (57.6)
Marital status Married Unmarried Widowed/Separated	656 (55.4) 347 (29.2) 182 (15.4)	Cultural compatibility Always Sometimes Never	888 (74.9) 269 (22.7) 28 (2.4)
Family setup Joint family system Nuclear	886 (74.8) 299 (25.2)	linguistic barriers Always Sometimes Never	192 (16.2) 351 (29.6) 642 (54.2)
Province of resettlement *Khyber Pakhtunkhwa* Haripur Mardan Nowshera Peshawar *Punjab* Mianwali	1104 (93.2) 396 (33.4) 075 (6.3) 239 (20.2) 394 (33.2) 81 (6.8)	Face discrimination Always Sometimes Never	190 (16.0) 361 (30.5) 634 (53.5)
		Social inclusion Always Sometimes Never	280 (23.6) 674 (56.9) 231 (19.5)
Mother Language Dari Pasthu other	463 (39.1) 599 (50.5) 123 (10.4)	Socioeconomic support Always Sometimes Never	884 (74.6) 242 (20.4) 59 (5.0)
Number of years in the host country <10 years 10–19 years >20 years	107 (9.0) 392 (33.1) 686 (57.9)	Access to clean water Always Sometimes Never	668 (56.4) 426 (35.9) 91 (7.7)
Residence Type Owner Rent Donor/Government supported	071 (6.0) 192 (16.2) 922 (77.8)	Access to Sanitation Always Sometimes Never	677 (57.1) 437 (36.9) 71 (6.0)
Place of residence Urban Rural	710 (59.9) 475 (40.1)	Access to education Always Sometimes Never	678 (57.2) 411 (34.7) 96 (8.1)
Level of education Uneducated <10 years of education 10–14 years of education 16 years of education and greater	429 (36.2) 647 (54.6) 99 (8.4) 10 (0.8)	Access to healthcare information Always Sometimes Never	82 (6.9) 403 (34) 700 (59.1)
Employment status Employed Unemployed	559 (47.2) 626 (52.8)	Access to healthcare Always Sometimes	586 (49.5) 599 (50.5)
Family monthly income <25,000 25,000–50,000 50,001–75,000 >75,001	826 (69.7) 241 (20.3) 84 (7.1) 34 (2.9)	Current Health Status Healthy Sick Chronic Health Illness Yes No	579 (48.9) 606 (51.1) 451 (38.1) 734 (61.9)
Self-rated socioeconomic status High Average Low	32 (2.7) 273 (23.0) 880 (74.3)	Last time visited the Health facility Within last 1 month Within last 1 year More than 1 year	334 (28.2) 394 (33.2) 457 (38.6)

**Table 2:** Descriptive statistics about health literacy among Afghan refugees in Pakistan (N = 1185).

Items	Pakistan *n* (%)	
	Yes	No
	(Often + Sometimes)	(Rarely + Not Applicable)
**Functional health literacy (FHL)**		
Need help to understand information	901 (76)	284 (24.0)
Easy to find someone for assistance	974 (82.2)	211 (17.8)
Need help for official documentation	948 (80)	237 (20)
**Communicative health literacy (COMHL)**		
Provide information for assistance	1100 (92.8)	85 (7.2)
Inquire about the queries	1046 (88.3)	139 (11.7)
Insist on explaining not clear content	1041 (87.8)	144 (12.2)
**Critical health literacy (CRHL)**		
Like to search facts related to health	578 (48.8)	607 (51.2)
Information appropriate for the situation	558 (47.1)	627 (52.9)
Evaluate the health care information reliability	504 (42.5)	681 (57.5)
Inspect the health care personnel’s advice based on your own research	558 (47.08)	627 (52.92)
**Empowerment related to health literacy (EMP)**		
Trust that there are ways for guiding government actions for health care	1079 (91)	106 (9)
Measure taken in last one year to tackle a health concern in community	495 (41.8)	690 (58.2)
More essential for every one health according to your point of view		
(a) Information and encouragement to lead healthy lifestyles	773 (65.2)	412 (34.8)
(b) Good housing, education, decent jobs and good local facilities	412 (34.8)	773 (65.2)

Abbreviations: FHL, functional health literacy; COMHL, communicative health literacy; CRHL, critical health literacy; EMP, empowerment-related health literacy.

**Table 3:** Logistic regression model of functional health literacy (FHL) and sociodemographic characteristics.

Sociodemographic Characteristics	*FHL 1*			*FHL 2*			*FHL 3*		
	*AOR*	95% CI for AOR		*AOR*	95% CI for AOR		*AOR*	95% CI for AOR	
		*Lower Bound*	*Upper Bound*		*Lower Bound*	*Upper Bound*		*Lower Bound*	*Upper Bound*
**Age**									
18–30 years	*Reference*			*Reference*			*Reference*		
31–45	0.85	0.58	1.26	0.94	0.6	1.48	0.95	0.62	1.46
46–60	0.79	0.55	1.12	0.87	0.58	1.3	1.02	0.69	1.49
61 years or older	0.77	0.42	1.4	0.48	0.25	0.9	0.94	0.49	1.78
**Gender**									
Male	*Reference*			*Reference*			*Reference*		
Female	1.2	0.912	1.587	1.22	0.931	1.271	1.72 *	1.269	2.322
**Marital status**									
Married	*Reference*			*Reference*			*Reference*		
Unmarried	1.02	0.74	1.41	1.15	0.79	1.66	0.7	0.5	0.98
Widowed/separated	1.2	0.78	1.83	1.31	0.81	2.12	1.3	0.81	2.11
**Family setup**									
Joint family system	*Reference*			*Reference*			*Reference*		
Nuclear	0.84	0.62	1.13	0.96	0.68	0.97	1.07	0.76	1.49
**Province of resettlement**									
Haripur	*Reference*			*Reference*			*Reference*		
Mardan	1.11	0.58	2.12	0.53	0.27	1.04	0.89	0.43	1.84
Nowshera	1.33	0.85	2.08	0.8	0.48	1.32	0.64	0.4	1.02
Peshawar	1.3	0.84	1.99	1.23	0.75	2.03	0.61	0.38	0.97
Kot Chandana Mianwali	1.12	0.58	2.16	0.51	0.25	1.03	0.68	0.34	1.38
**Number of years in the host country**									
<10 years	*Reference*			*Reference*			*Reference*		
10–19 years	0.92	0.54	1.57	1.19	0.63	2.25	0.5	0.26	0.95
>20 years	0.95	0.57	1.58	0.87	0.47	1.58	0.59	0.32	1.11
**Mother Tongue**									
Dari	*Reference*			*Reference*			*Reference*		
Pashto	0.84	0.62	1.13	1.27	0.9	1.79	1.01	0.72	1.4
other	1.43	0.84	2.42	1.3	0.73	2.31	0.76	0.45	1.27
**Residence type**									
Owner	*Reference*			*Reference*			*Reference*		
Government/donor-supported	0.5	0.25	1	1.22	0.62	2.39	1.84	0.96	3.53
Rent	0.61	0.3	1.24	1.46	0.73	2.91	1.15	0.6	2.22
**Place of residence**									
Urban	*Reference*			*Reference*			*Reference*		
Rural	1.18	0.89	1.57	1.21	0.89	1.22	1.06	0.79	1.43
**Level of Education**									
Uneducated	*Reference*			*Reference*			*Reference*		
<10 years of age	0.92	0.68	1.26	0.95	0.66	1.35	0.65	0.46	0.91
10–14 years of age	0.72	0.42	1.22	0.82	0.44	1.54	0.98	0.53	1.84
16 years of education and greater	0.64	0.15	2.71	0.41	0.09	1.83	0.38	0.09	1.7
Family monthly income (in Pakistani rupees *)									
<25,000	*Reference*			*Reference*			*Reference*		
25,000–50,000	0.85	0.59	1.22	0.83	0.55	1.24	1.41	0.93	2.14
50,001–75,000	0.62	0.36	1.06	0.78	0.41	1.47	1.04	0.57	1.9
>75,001	0.38	0.17	0.82	1.33	0.51	3.51	1.67	0.64	4.37
**Self-rated socioeconomic status**									
Upper class	*Reference*			*Reference*			*Reference*		
Middle class	1.13	0.48	2.66	0.78	0.26	2.31	1.01	0.40	2.57
Lower class	1.56	0.68	3.57	0.55	0.19	1.57	1.17	0.48	2.88
**Smoking**									
Frequently	*Reference*			*Reference*			*Reference*		
Occasionally	0.75	0.481	1.17	1	0.6	1.69	0.86	0.53	1.4
Non-smokers	0.85	0.62	1.17	0.88	0.61	1.27	0.91	0.64	1.29
**Substance abuse (Naswar)**									
Frequently	*Reference*			*Reference*			*Reference*		
Occasionally	0.99	0.62	1.58	0.94	0.56	1.59	0.68	0.41	1.13
Non-users	1.09	0.76	1.55	1.13	0.77	1.68	0.73	0.49	1.08
**Cultural compatibility in the host country**									
Always	*Reference*			*Reference*			*Reference*		
Sometimes	0.93	0.66	1.3	0.85	0.58	1.24	1.39	0.95	2.03
Never	0.63	0.26	1.52	0.92	0.32	2.61	0.85	0.33	2.2
**linguistic barriers in the host country**									
Always	*Reference*			*Reference*			*Reference*		
Sometimes	0.84	0.54	1.29	0.96	0.57	1.6	1.57	0.97	2.53
Never	1.15	0.77	1.73	0.86	0.53	1.39	1.07	0.7	1.63
**Face discrimination as a refugee in the host country**									
Always	*Reference*			*Reference*			*Reference*		
Sometimes	0.73	0.47	1.16	0.57	0.32	1.01	0.8	0.48	1.34
Never	0.78	0.5	1.22	0.45	0.26	0.78	0.78	0.47	1.27
**Social inclusion as a refugee in the host country**									
Always	*Reference*			*Reference*			*Reference*		
Sometimes	0.99	0.69	1.41	1.23	0.84	1.8	0.79	0.54	1.17
Never	1.09	0.7	1.69	1.73	1.04	2.89	0.8	0.49	1.3
**Socioeconomic support from government or donor agencies**									
Always	*Reference*			*Reference*			*Reference*		
Sometimes	0.98	0.68	1.4	0.74	0.5	1.1	1.27	0.85	1.9
Never	0.79	0.41	1.51	0.64	0.31	1.31	1.73	0.83	3.64
**Access to clean water**									
Always	*Reference*			*Reference*			*Reference*		
Sometimes	2.64	0.8	8.7	12.09	1.34	108.8	1.9	0.58	6.23
Never	1.31	0.32	5.29	0.89	0.2	3.97	0.83	0.20	3.39
**Access to sanitation**									
Always	*Reference*			*Reference*			*Reference*		
Sometimes	0.95	0.7	1.28	1.37	0.97	1.94	0.81	0.58	1.12
Never	0.94	0.5	1.76	0.95	0.49	1.83	1.2	0.61	2.36
**Access to Education**									
Always	*Reference*			*Reference*			*Reference*		
Sometimes	1.29	0.95	1.76	0.92	0.66	1.3	0.81	0.59	1.13
Never	0.73	0.44	1.2	0.97	0.54	1.75	0.97	0.55	1.72
**Access to health care information**									
Never	*Reference*			*Reference*			*Reference*		
Sometimes	0.36	0.11	1.19	0.07	0.01	0.6	0.53	0.16	1.73
Always	0.83	0.195	3.53	0.49	0.1	2.29	0.98	0.23	4.24
**Access to healthcare**									
Always	*Reference*			*Reference*			*Reference*		
Sometimes	0.87	0.66	1.14	1.22	0.91	1.23	1.06	0.80	1.42
**Current Health Status**									
Healthy	*Reference*			*Reference*			*Reference*		
Sick	1.01	0.67	1.54	1.27	0.78	1.26	1.1	0.69	1.75
**Chronic Health Illness**									
Yes	*Reference*			*Reference*			*Reference*		
No	1.06	0.69	1.63	0.98	0.59	0.97	0.82	0.51	1.31
**Last time visited Health facility**									
Within the last 1 month	*Reference*			*Reference*			*Reference*		
Within the last 1 year	0.87	0.6	1.22	1.17	0.77	1.76	1.22	0.83	1.79
More than 1 year	1.02	0.72	1.44	0.94	0.64	1.4	1.12	0.78	1.62

Abbreviations: FHL, functional health literacy; COMHL, communicative health literacy; CRHL, critical health literacy; EMP, empowerment-related health literacy; AOR, adjusted odds ratio; CI, confidence interval. Note: * indicates that the *p*-value is significant at *p* ≤ 0.05.

**Table 4:** Logistic regression model of communicative health literacy (COMHL) and sociodemographic characteristics.

Sociodemographic Characteristics	*COMHL 1*			*COMHL 2*			*COMHL 3*		
	*AOR*	95% CI for AOR		*AOR*	95% CI for AOR		*AOR*	95% CI for AOR	
		*Lower Bound*	*Upper Bound*		*Lower Bound*	*Upper Bound*		*Lower Bound*	*Upper Bound*
**Age**									
18–30 years	*Reference*			*Reference*			*Reference*		
31–45	1.46	0.77	2.77	1.07	0.65	1.78	1.14	0.67	1.93
46–60	1.36	0.77	2.40	1.34	0.84	2.14	0.89	0.56	1.41
61 years or older	2.25	0.69	7.39	0.75	0.36	1.56	1.25	0.54	2.92
**Gender**									
Male	*Reference*			*Reference*			*Reference*		
Female	1.11	0.70	1.77	0.84	0.58	1.21	0.82	0.57	1.19
**Marital status**									
Married	*Reference*			*Reference*			*Reference*		
\Unmarried	1.59	0.90	2.81	0.79	0.52	1.21	0.86	0.56	1.31
Widowed/separated	1.12	0.56	2.24	0.84	0.49	1.45	1.14	0.64	2.04
**Family setup**									
Joint family system	*Reference*			*Reference*			*Reference*		
Nuclear	1.49	0.85	2.62	1.05	0.70	1.59	1.30	0.85	1.99
**Province of resettlement**									
Haripur	*Reference*			*Reference*			*Reference*		
Mardan	0.75	0.27	2.04	0.66	0.28	1.58	3.27 *	1.06	10.12
Nowshera	1.39	0.61	3.16	0.89	0.48	1.66	1.19	0.67	2.11
Peshawar	0.65	0.31	1.35	0.68	0.38	1.24	1.82 *	1.00	3.32
Kot Chandana Mianwali	0.95	0.30	3.00	0.63	0.27	1.46	0.59	0.29	1.23
**Number of years in the host country**									
<10 years	*Reference*			*Reference*			*Reference*		
10–19 years	1.77	0.78	4.03	1.09	0.54	2.20	1.06	0.53	2.15
>20 years	1.99	0.91	4.38	1.12	0.58	2.19	1.16	0.59	2.27
**Mother Tongue**									
Dari	*Reference*			*Reference*			*Reference*		
Pashto	1.40	0.85	2.31	1.12	0.75	1.67	0.86	0.57	1.29
other	1.65	0.67	4.08	1.59	0.79	3.20	1.49	0.70	3.18
**Residence type**									
Owner	*Reference*			*Reference*			*Reference*		
Government/donor-supported	0.58	0.16	2.04	0.11	0.02	0.85	1.40	0.60	3.29
Rent	0.57	0.16	2.07	0.13	0.02	0.98	0.99	0.43	2.32
**Place of residence**									
Urban	*Reference*			*Reference*			*Reference*		
Rural	1.27	0.79	2.04	1.06	0.73	1.54	0.69 *	0.48	0.99
**Level of Education**									
Uneducated	*Reference*			*Reference*			*Reference*		
<10 years of age	1.07	0.63	1.79	0.94	0.62	1.42	1.27	0.84	1.90
10–14 years of age	1.09	0.45	2.64	0.73	0.37	1.44	1.17	0.53	2.58
16 years of education and greater	1.05	0.11	10.18	0.79	0.08	7.40	0.38	0.07	2.18
Family monthly income (in Pakistani rupees *)									
<25,000	*Reference*			*Reference*			*Reference*		
25,000–50,000	1.65	0.85	3.21	1.02	0.63	1.67	1.08	0.66	1.78
50,001–75,000	0.83	0.36	1.95	1.45	0.65	3.24	0.69	0.33	1.43
>75,001	0.48	0.14	1.59	0.78	0.27	2.23	1.10	0.30	3.98
**Self-rated socioeconomic status**									
Upper class	*Reference*			*Reference*			*Reference*		
Middle class	3.85	1.16	12.77	1.21	0.37	4.02	1.28	0.38	4.30
Lower class	3.88	1.25	12.06	1.19	0.37	3.77	1.28	0.40	4.16
**Smoking**									
Frequently	*Reference*			*Reference*			*Reference*		
Occasionally	1.05	0.49	2.23	0.82	0.46	1.46	1.18	0.62	2.24
Non-smokers	1.35	0.80	2.29	0.98	0.64	1.51	0.96	0.62	1.48
**Substance abuse (Naswar)**									
Frequently	*Reference*			*Reference*			*Reference*		
Occasionally	2.80	1.07	7.35	0.88	0.48	1.60	2.93 *	1.39	6.17
Non-users	0.98	0.55	1.73	0.90	0.56	1.44	1.81 *	1.15	2.85
**Cultural compatibility in the host country**									
Always	*Reference*			*Reference*			*Reference*		
Sometimes	1.41	0.78	2.55	0.84	0.55	1.30	0.98	0.63	1.51
Never	1.00	0.22	4.62	0.97	0.27	3.46	1.86	0.41	8.43
**linguistic barriers in the host country**									
Always	*Reference*			*Reference*			*Reference*		
Sometimes	0.65	0.28	1.50	1.02	0.57	1.82	1.18	0.67	2.07
Never	0.56	0.26	1.21	1.12	0.65	1.92	1.25	0.74	2.12
**Face discrimination as a refugee in the host country**									
Always	*Reference*			*Reference*			*Reference*		
Sometimes	0.76	0.34	1.71	0.95	0.52	1.74	0.84	0.48	1.48
Never	0.69	0.32	1.52	1.20	0.66	2.17	1.12	0.64	1.98
**Social inclusion as a refugee in the host country**									
Sometimes	*Reference*			*Reference*			*Reference*		
Always	0.73	0.39	1.35	0.97	0.61	1.54	0.74	0.44	1.23
Never	0.77	0.36	1.65	1.76	0.93	3.33	0.56	0.31	1.01
**Socioeconomic support from government or donor agencies**									
Always	*Reference*			*Reference*			*Reference*		
Sometimes	1.50	0.77	2.93	1.23	0.74	2.02	1.02	0.64	1.63
Never	0.78	0.27	2.25	0.61	0.29	1.31	1.14	0.43	3.06
**Access to clean water**									
Always	*Reference*			*Reference*			*Reference*		
Sometimes	0.71	0.19	2.63	0.69	0.22	2.13	0.54	0.18	1.61
Never	4.32	0.16	120.12	1.16	0.21	6.41	1.75	0.20	15.09
**Access to sanitation**									
Always	*Reference*			*Reference*			*Reference*		
Sometimes	1.01	0.61	1.65	0.76	0.52	1.13	0.96	0.65	1.43
Never	1.96	0.56	6.87	1.46	0.54	3.92	1.40	0.59	3.31
**Access to Education**									
Always	*Reference*				*Reference*		*Reference*		
Sometimes	1.15	0.69	1.91	0.59	0.40	0.88	1.25	0.83	1.87
Never	1.49	0.55	4.05	0.92	0.44	1.93	1.38	0.64	2.94
**Access to health care information**									
Never	*Reference*			*Reference*			*Reference*		
Sometimes	1.12	0.30	4.14	1.30	0.42	4.07	1.16	0.39	3.43
Always	0.82	0.03	22.92	0.95	0.16	5.69	0.44	0.05	3.85
**Access to healthcare**									
Always	*Reference*			*Reference*			*Reference*		
Sometimes	1.27	0.81	1.98	1.01	0.71	1.45	0.75	0.52	1.07
**Current Health Status**									
Healthy	*Reference*			*Reference*			*Reference*		
Sick	1.11	0.52	2.37	1.09	0.61	1.94	1.82	0.96	3.45
**Chronic Health Illness**									
Yes	Reference			Reference			Reference		
No	0.64	0.30	1.37	0.76	0.42	1.37	0.62	0.32	1.20
**Last time visited Health facility**									
Within the last 1 month	*Reference*			*Reference*			*Reference*		
Within the last 1 year	0.91	0.51	1.65	1.25	0.77	2.02	0.82	0.51	1.30
More than 1 year	1.08	0.61	1.92	1.08	0.69	1.70	1.25	0.78	2.01

Note: * indicates that the *p* value is significant at *p* ≤ 0.05.

**Table 5:** Logistic regression model of critical health literacy (CRHL) and sociodemographic characteristics.

Sociodemographic Characteristics	*CRHL 1*			*CRHL 2*			*CRHL 3*			*CRHL 4*		
	*AOR*	95% CI for AOR		*AOR*	95% CI for AOR		*AOR*	95% CI for AOR		*AOR*	95% CI for AOR	
		*Lower Bound*	*Upper Bound*		*Lower Bound*	*Upper Bound*		*Lower Bound*	*Upper Bound*		*Lower Bound*	*Upper Bound*
**Age**												
18–30 years	*Reference*			*Reference*			*Reference*			*Reference*		
31–45	1.23	0.72	2.08	1.19	0.67	2.12	1.06	0.60	1.86	1.09	0.64	1.84
46–60	1.35	0.83	2.19	1.35	0.81	2.24	0.62	0.39	1.01	1.08	0.68	1.73
61 years or older	0.65	0.30	1.40	1.81	0.76	4.33	1.10	0.48	2.48	0.42	0.22	0.82
**Gender**												
Male	*Reference*			*Reference*			*Reference*			*Reference*		
Female	0.46 *	0.31	0.69	0.85	0.67	1.08	1.61 *	1.10	2.35	1.23	0.86	1.77
**Marital status**												
Married	*Reference*			*Reference*			*Reference*			*Reference*		
Unmarried	1.46	0.92	2.31	1.15	0.71	1.85	1.02	0.66	1.58	0.81	0.53	1.23
Widowed/separated	2.22 *	1.14	4.32	1.06	0.59	1.91	0.89	0.51	1.53	0.78	0.46	1.31
**Family setup**												
Joint family system	*Reference*			*Reference*			*Reference*			*Reference*		
Nuclear	1.21	0.77	1.89	0.97	0.74	1.26	0.68	0.46	1.00	0.98	0.66	1.46
**Province of resettlement**												
Haripur	*Reference*			*Reference*			*Reference*			*Reference*		
Mardan	1.39	0.56	3.47	0.79	0.32	1.96	5.38 *	1.76	16.48	1.34	0.59	3.04
Nowshera	1.50	0.78	2.88	1.82	0.84	3.96	1.79 *	1.03	3.10	1.49	0.86	2.57
Peshawar	0.95	0.51	1.77	0.92	0.49	1.72	2.48 *	1.41	4.36	1.76	1.00	3.08
Kot Chandana Mianwali	1.53	0.58	4.05	1.00	0.35	2.86	4.54 *	1.60	12.85	5.73 *	1.85	17.77
**Number of years in the host country**												
<10 years	*Reference*			*Reference*			*Reference*			*Reference*		
10–19 years	0.83	0.39	1.75	1.69	0.80	3.58	0.72	0.34	1.51	0.77	0.39	1.53
>20 years	1.00	0.49	2.07	1.58	0.77	3.22	0.85	0.41	1.73	0.96	0.50	1.87
**Mother Tongue**												
Dari	*Reference*			*Reference*			*Reference*			*Reference*		
Pashto	1.47	0.94	2.26	1.09	0.69	1.72	1.46	0.97	2.19	1.23	0.83	1.82
Other	1.06	0.56	2.03	0.68	0.35	1.32	1.08	0.57	2.07	0.92	0.49	1.71
**Residence type**												
Owner	*Reference*			*Reference*			*Reference*			*Reference*		
Government/donor-supported	1.72	0.76	3.89	0.86	0.34	2.21	0.77	0.33	1.79	1.45	0.65	3.26
Rent	1.58	0.68	3.62	0.90	0.35	2.36	1.15	0.49	2.70	0.74	0.34	1.65
**Place of residence**												
Urban	*Reference*			*Reference*			*Reference*			*Reference*		
Rural	1.11	0.75	1.65	1.03	0.81	1.31	1.02	0.71	1.48	0.85	0.60	1.21
**Level of Education**												
Uneducated	*Reference*			*Reference*			*Reference*			*Reference*		
<10 years of age	1.37	0.90	2.03	0.85	0.53	1.34	0.78	0.51	1.18	0.90	0.60	1.35
10–14 years of age	1.08	0.52	2.22	0.67	0.32	1.39	1.14	0.51	2.55	0.80	0.39	1.62
≥ 16 years education	3.31	0.00	2.09	2.26	0.00	1.11	1.53	0.00	2.22	0.29	0.05	1.57
Family monthly income (in Pakistani rupees *)												
<25,000	*Reference*			*Reference*			*Reference*			*Reference*		
25,000–50,000	0.89	0.54	1.46	0.72	0.44	1.18	1.04	0.64	1.68	0.77	0.49	1.23
50,001–75,000	1.21	0.54	2.75	0.79	0.37	1.70	2.30	0.79	6.70	0.70	0.34	1.44
>75,001	0.96	0.28	3.23	0.74	0.23	2.39	2.32	0.51	10.57	1.02	0.32	3.24
**Self-rated socioeconomic status**												
Upper class	*Reference*			*Reference*			*Reference*			*Reference*		
Middle class	1.84	0.62	5.49	2.18	0.55	8.59	0.65	0.17	2.49	0.81	0.21	3.18
Lower class	2.27	0.80	6.45	1.31	0.35	4.85	0.54	0.15	1.95	0.58	0.15	2.19
**Smoking**												
Frequently	*Reference*			*Reference*			*Reference*			*Reference*		
Occasionally	0.87	0.46	1.64	1.58	0.83	2.99	1.21	0.65	2.26	0.84	0.47	1.51
Non-smokers	0.77	0.49	1.21	2.30	1.45	3.64	1.21	0.80	1.85	0.88	0.58	1.34
**Substance abuse (Naswar)**												
Frequently	*Reference*			*Reference*			*Reference*			*Reference*		
Occasionally	1.45	0.75	2.79	0.79	0.42	1.50	1.60	0.78	3.30	0.84	0.43	1.62
Non-users	1.46	0.90	2.35	0.96	0.58	1.61	1.18	0.74	1.91	0.86	0.53	1.40
**Cultural compatibility in the host country**												
Always	*Reference*			*Reference*			*Reference*			*Reference*		
Sometimes	0.95	0.60	1.51	0.93	0.57	1.51	0.92	0.59	1.45	1.02	0.66	1.59
Never	3.69	0.47	28.92	0.27	0.09	0.76	0.82	0.27	2.45	1.67	0.46	6.13
**linguistic barriers in the host country**												
Always	*Reference*			*Reference*			*Reference*			*Reference*		
Sometimes	0.90	0.50	1.63	1.33	0.68	2.58	1.46	0.83	2.57	0.91	0.51	1.62
Never	0.97	0.55	1.69	1.01	0.56	1.82	1.36	0.82	2.27	0.85	0.50	1.44
**Face discrimination as a refugee in the host country**												
Always	*Reference*			*Reference*			*Reference*			*Reference*		
Sometimes	0.76	0.42	1.40	0.93	0.48	1.78	0.77	0.43	1.38	0.87	0.50	1.51
Never	0.87	0.47	1.62	1.25	0.65	2.42	0.81	0.45	1.44	0.98	0.57	1.69
**Social inclusion as a refugee in the host country**												
Sometimes	*Reference*			*Reference*			*Reference*			*Reference*		
Always	0.99	0.60	1.62	1.21	0.73	2.00	1.98	1.27	3.08	0.80	0.49	1.31
Never	0.92	0.50	1.70	1.45	0.76	2.77	1.98	1.11	3.53	0.52	0.29	0.93
**Socioeconomic support from the government or donor agencies**												
Always	*Reference*			*Reference*			*Reference*			*Reference*		
Sometimes	0.96	0.58	1.58	1.05	0.62	1.80	0.74	0.47	1.16	0.80	0.51	1.25
Never	0.46	0.20	1.03	0.33	0.15	0.72	0.74	0.31	1.76	1.67	0.60	4.64
**Access to clean water**												
Always	*Reference*			*Reference*			*Reference*			*Reference*		
Sometimes	1.33	0.31	5.67	0.71	0.19	2.63	0.66	0.17	2.57	0.53	0.15	1.87
Never	0.63	0.13	3.18	0.49	0.07	3.68	0.49	0.08	2.98	0.47	0.09	2.37
**Access to sanitation**												
Always	*Reference*			*Reference*			*Reference*			*Reference*		
Sometimes	1.26	0.82	1.93	0.95	0.62	1.47	1.21	0.80	1.82	0.95	0.64	1.41
Never	1.12	0.49	2.58	2.78	0.79	9.71	1.24	0.54	2.82	0.83	0.39	1.76
**Access to Education**												
Always	*Reference*			*Reference*			*Reference*			*Reference*		
Sometimes	1.13	0.74	1.72	1.35	0.86	2.13	1.14	0.76	1.72	0.70	0.48	1.03
Never	1.10	0.52	2.34	1.56	0.69	3.56	1.19	0.58	2.41	0.97	0.46	2.03
**Access to health care information**												
Never	*Reference*			*Reference*			*Reference*			*Reference*		
Sometimes	0.91	0.21	3.90	1.63	0.44	6.13	1.16	0.30	4.51	1.97	0.56	6.94
Always	1.93	0.34	10.82	2.89	0.34	24.77	2.00	0.31	13.08	3.26	0.57	18.73
**Access to healthcare**												
Always	*Reference*			*Reference*			*Reference*			*Reference*		
Sometimes	1.46 *	1.00	2.12	1.04	0.83	1.32	1.29	0.90	1.85	1.21	0.85	1.71
**Current Health Status**												
Healthy	*Reference*			*Reference*			*Reference*			*Reference*		
Sick	2.16 *	1.05	4.47	0.60 *	0.41	0.85	1.16	0.65	2.05	0.97	0.56	1.68
**Chronic Health Illness**												
Yes	*Reference*			*Reference*			*Reference*			*Reference*		
No	0.47 *	0.22	0.98	1.25	0.86	1.81	0.96	0.53	1.72	0.91	0.52	1.58
**Last time visited Health facility**												
Within the last 1 month	*Reference*			*Reference*			*Reference*			*Reference*		
Within the last 1 year	1.08	0.66	1.77	0.70	0.42	1.17	0.90	0.55	1.45	1.03	0.65	1.62
More than 1 year	1.18	0.73	1.90	1.25	0.74	2.12	0.96	0.60	1.53	1.37	0.87	2.15

Note: * indicates that the *p* value is significant at *p* ≤ 0.05.

**Table 6:** Logistic regression model of empowerment related to health literacy (EMP) and sociodemographic characteristics.

Sociodemographic Characteristics	*EMP 1*			*EMP 2*			*EMP 3*			*EMP 4*		
	*AOR*	95% CI for AOR		*AOR*	95% CI for AOR		*AOR*	95% CI for AOR		*AOR*	95% CI for AOR	
		*Lower Bound*	*Upper Bound*		*Lower Bound*	*Upper Bound*		*Lower Bound*	*Upper Bound*		*Lower Bound*	*Upper Bound*
**Age**												
18–30 years	*Reference*			*Reference*			*Reference*			*Reference*		
31–45	0.82	0.45	1.51	0.84	0.59	1.19	1.20	0.84	1.70	0.84	0.59	1.19
46–60	0.91	0.52	1.58	0.88	0.65	1.21	1.13	0.83	1.55	0.88	0.65	1.21
61 years or older	0.47	0.21	1.02	1.27	0.74	2.18	0.79	0.46	1.35	1.27	0.74	2.18
**Gender**												
Male	*Reference*			*Reference*			*Reference*			*Reference*		
Female	0.98	0.65	1.49	0.96	0.75	1.22	1.16	0.90	1.49	0.85	0.669	1.100
**Marital status**												
Married	*Reference*			*Reference*			*Reference*			*Reference*		
Unmarried	1.22	0.73	2.02	0.95	0.71	1.27	1.06	0.79	1.41	0.95	0.71	1.27
Widowed/separated	1.08	0.59	1.99	0.74	0.51	1.09	1.34	0.92	1.97	0.74	0.51	1.09
**Family setup**												
Joint family system	*Reference*			*Reference*			*Reference*			*Reference*		
Nuclear	0.83	0.53	1.30	0.73 *	0.56	0.95	1.02	0.78	1.35	0.97	0.73	1.282
**Province of resettlement**												
Haripur	*Reference*			*Reference*			*Reference*			*Reference*		
Mardan	0.40 *	0.17	0.93	0.75	0.40	1.41	1.33	0.71	2.49	0.75	0.40	1.41
Nowshera	1.34	0.63	2.84	1.32	0.88	1.96	0.76	0.51	1.13	1.32	0.88	1.96
Peshawar	0.86	0.45	1.64	1.18	0.80	1.75	0.85	0.57	1.25	1.18	0.80	1.75
Kot Chandana Mianwali	0.52	0.21	1.31	1.99 *	1.13	3.52	0.50	0.28	0.89	1.99 *	1.13	3.52
**Number of years in the host country**												
<10 years	*Reference*			*Reference*			*Reference*			*Reference*		
10–19 years	1.25	0.58	2.72	1.23	0.75	2.02	0.81	0.50	1.34	1.23	0.75	2.02
>20 years	1.22	0.59	2.55	1.29	0.80	2.07	0.78	0.48	1.25	1.29	0.80	2.07
**Mother Tongue**												
Dari	*Reference*			*Reference*			*Reference*			*Reference*		
Pashto	0.90	0.56	1.44	0.86	0.65	1.14	1.16	0.88	1.53	0.86	0.65	1.14
other	0.74	0.36	1.49	1.41	0.91	2.19	0.71	0.46	1.10	1.41	0.91	2.19
**Residence type**												
Owner	*Reference*			*Reference*			*Reference*			*Reference*		
Government/donor-supported	0.97	0.40	2.38	1.01	0.56	1.81	0.99	0.55	1.79	1.01	0.56	1.81
Rent	1.35	0.54	3.39	0.93	0.51	1.69	1.08	0.59	1.95	0.93	0.51	1.69
**Place of residence**												
Urban	*Reference*			*Reference*			*Reference*			*Reference*		
Rural	1.02	0.67	1.55	1.06 *	5.83	1.35	1.37 *	1.07	1.76	0.72 *	0.57	0.93
**Level of Education**												
uneducated	*Reference*			*Reference*			*Reference*			*Reference*		
<10 years of age	1.88 *	1.18	2.99	0.93	0.70	1.23	1.08	0.82	1.43	0.93	0.70	1.23
10–14 years of age	0.97	0.47	2.00	1.40	0.86	2.27	0.72	0.44	1.16	1.40	0.86	2.27
16 years of education and greater	1.26	0.14	11.17	1.18	0.29	4.88	0.85	0.20	3.49	1.18	0.29	4.88
**Family monthly income (in Pakistani rupees)**												
<25,000	*Reference*			*Reference*			*Reference*			*Reference*		
25,000–50,000	0.91	0.54	1.54	1.00	0.72	1.40	1.00	0.71	1.39	1.00	0.72	1.40
50,001–75,000	0.95	0.41	2.16	1.04	0.63	1.72	0.96	0.58	1.60	1.04	0.63	1.72
>75,001	1.06	0.29	3.90	0.61	0.26	1.44	1.64	0.69	3.87	0.61	0.26	1.44
**Self-rated socioeconomic status**												
Upper class	*Reference*			*Reference*			*Reference*			*Reference*		
Middle class	2.02	0.65	6.35	2.12	0.80	5.64	0.47	0.18	1.25	2.12	0.80	5.64
Lower class	2.40	0.80	7.23	2.54	0.98	6.56	0.39	0.15	1.02	2.54	0.98	6.56
**Smoking**												
Frequently	*Reference*			*Reference*			*Reference*			*Reference*		
Occasionally	1.52	0.74	3.10	1.29	0.85	1.96	0.78	0.51	1.18	1.29	0.85	1.96
Non-smokers	1.26	0.79	2.02	1.27	0.95	1.70	0.79	0.59	1.05	1.27	0.95	1.70
**Substance abuse (Naswar)**												
Frequently	*Reference*			*Reference*			*Reference*			*Reference*		
Occasionally	1.01	0.49	2.09	1.34	0.88	2.05	0.75	0.49	1.14	1.34	0.88	2.05
Non-users	0.81	0.48	1.38	0.87	0.63	1.19	1.15	0.84	1.59	0.87	0.63	1.19
**Cultural compatibility in the host country**												
Always	*Reference*			*Reference*			*Reference*			*Reference*		
Sometimes	1.04	0.62	1.74	1.50 *	1.11	2.02	0.67 *	0.50	0.90	1.50 *	1.11	2.02
Never	0.96	0.21	4.35	0.95	0.41	2.24	1.05	0.45	2.47	0.95	0.41	2.24
**linguistic barriers in the host country**												
Always	*Reference*			*Reference*			*Reference*			*Reference*		
Sometimes	1.56	0.85	2.88	0.87	0.58	1.29	1.15	0.77	1.72	0.87	0.58	1.29
Never	1.75	1.00	3.07	1.03	0.71	1.49	0.97	0.67	1.40	1.03	0.71	1.49
**Face discrimination as a refugee in the host country**												
Always	*Reference*			*Reference*			*Reference*			*Reference*		
Sometimes	1.27	0.66	2.44	1.18	0.79	1.76	0.85	0.57	1.26	1.18	0.79	1.76
Never	1.28	0.67	2.42	0.94	0.63	1.39	1.07	0.72	1.59	0.94	0.63	1.39
**Social inclusion as a refugee in the host country**												
Sometimes	*Reference*			*Reference*			*Reference*			*Reference*		
Always	0.71	0.41	1.25	1.09	0.79	1.51	0.92	0.66	1.27	1.09	0.79	1.51
Never	0.94	0.46	1.92	1.36	0.91	2.03	0.73	0.49	1.10	1.36	0.91	2.03
**Socioeconomic support from the government or donor agencies**												
Always	*Reference*			*Reference*			*Reference*			*Reference*		
Sometimes	1.04	0.61	1.79	0.86	0.62	1.20	1.16	0.84	1.61	0.86	0.62	1.20
Never	1.54	0.49	4.83	1.06	0.59	1.91	0.94	0.52	1.70	1.06	0.59	1.91
**Access to clean water**												
Always	*Reference*			*Reference*			*Reference*			*Reference*		
Sometimes	1.93	0.37	10.18	1.12	0.48	2.64	0.89	0.38	2.10	1.12	0.48	2.64
Never	1.15	0.14	9.59	0.32	0.08	1.27	3.12	0.78	12.38	0.32	0.08	1.27
**Access to sanitation**												
Always	*Reference*			*Reference*			*Reference*			*Reference*		
Sometimes	0.77	0.49	1.20	1.20	0.92	1.58	0.83	0.63	1.09	1.20	0.92	1.58
Never	1.12	0.41	3.11	1.82	1.06	3.13	0.55	0.32	0.94	1.82	1.06	3.13
**Access to Education**												
Always	*Reference*			*Reference*			*Reference*			*Reference*		
Sometimes	1.07	0.68	1.70	1.10	0.83	1.44	0.91	0.69	1.20	1.10	0.83	1.44
Never	0.96	0.43	2.15	1.34	0.84	2.13	0.75	0.47	1.19	1.34	0.84	2.13
**Access to health care information**												
Never	*Reference*			*Reference*			*Reference*			*Reference*		
Sometimes	0.58	0.11	3.09	0.62	0.26	1.46	1.62	0.68	3.84	0.62	0.26	1.46
Always	0.87	0.09	8.09	2.21	0.54	9.11	0.45	0.11	1.86	2.21	0.54	9.11
**Access to healthcare**												
Always	*Reference*			*Reference*			*Reference*			*Reference*		
Sometimes	0.97	0.65	1.45	0.90	0.71	1.14	0.81	0.64	1.03	1.22	0.96	1.56
**Current Health Status**												
Healthy	*Reference*			*Reference*			*Reference*			*Reference*		
Sick	1.01	0.53	1.93	0.70	0.49	1.01	0.97	0.67	1.42	1.02	0.70	1.49
**Chronic Health Illness**												
Yes	*Reference*			*Reference*			*Reference*			*Reference*		
No	0.85	0.44	1.63	1.56 *	1.08	2.26	1.04	0.70	1.53	0.95	0.65	1.414
**Last time visited Health facility**												
Within the last 1 month	*Reference*			*Reference*			*Reference*			*Reference*		
Within the last 1 year	0.84	0.48	1.47	0.89	0.64	1.24	1.12	0.81	1.56	0.89	0.64	1.24
More than 1 year	0.81	0.47	1.38	1.19	0.87	1.62	0.84	0.62	1.15	1.19	0.87	1.62

Note: * indicates that the *p* value is significant at *p* ≤ 0.05.

## Data Availability

Due to ethical and regulatory constraints, the full dataset cannot be made publicly available. However, the authors confirm that reasonable requests for access to de-identified data necessary to verify the results will be considered by the corresponding author, subject to institutional approval and compliance with the original IRB conditions.
